# Research on the Effect of Load and Rotation Speed on Resistance to Combined Wear of Stainless Steels Using ANOVA Analysis

**DOI:** 10.3390/ma16124284

**Published:** 2023-06-09

**Authors:** Goran Rozing, Miroslav Duspara, Branislav Dudic, Borislav Savkovic

**Affiliations:** 1 Faculty of Electrical Engineering, Computer Science and Information Technology, University of Osijek, 31000 Osijek, Croatia; goran.rozing@ferit.hr; 2 Mechanical Engineering Faculty in Slavonski Brod, University of Slavonski Brod, 35000 Slavonski Brod, Croatia; mduspara@unisb.hr; 3 Faculty of Management, Comenius University Bratislava, 81499 Bratislava, Slovakia; 4 Faculty of Economics and Engineering Management, University Business Academy, 21000 Novi Sad, Serbia; 5 Faculty of Technical Sciences, University of Novi Sad, 21000 Novi Sad, Serbia

**Keywords:** tribocorrosion, stainless steels, induction hardening, load, rotation speed, wear

## Abstract

This research was carried out with the aim of obtaining appropriate principles for describing the influence of working parameters and the aggressive action of an acidic medium on the wear and corrosion resistance of martensitic stainless steels. Tribological tests were performed on induction-hardened surfaces of stainless steels X20Cr13 and X17CrNi16-2 under combined wear conditions at a load of 100 to 300 N and a rotation speed of 382 to 754 min^−1^. The wear test was carried out on a tribometer with the use of an aggressive medium in the chamber. After each wear cycle on the tribometer, the samples were exposed to corrosion action in a corrosion test bath. Analysis of variance revealed a significant influence of rotation speed and load due to wear on the tribometer. Testing the difference in the mass loss values of the samples due to corrosion using the Mann–Whitney U test did not show a significant effect of corrosion. Steel X20Cr13 showed greater resistance to combined wear, which had a 27% lower wear intensity compared to steel X17CrNi16-2. The increase in wear resistance of X20Cr13 steel can be attributed to the higher surface hardness achieved and the effective depth of hardening. The mentioned resistance is the result of the creation of a martensitic surface layer with dispersed carbides, which increases the resistance to abrasion, dynamic durability, and fatigue of the surface of the protective layer.

## 1. Introduction

The problems of friction and wear in practice are very complex due to the unfolding of various tribological processes. Especially in the process industry, e.g., food and petrochemical, there are frequent examples of complex wear, where the materials in contact are not only exposed to mechanical wear but can also be influenced by a corrosive environment. Under such conditions, the wear intensity of tribological contacts cannot be simply predicted based on wear resistance without corrosion or corrosion resistance in the absence of friction. The reason for this is that the mentioned parameters are not independent of each other, and their synergistic effect can significantly increase the rate of material degradation [1,2]. This phenomenon can be especially pronounced in those systems that have not been in use for a long time, due to occasional work, long-term transport, storage, etc. In such conditions, if adequate protection measures are not taken, corrosion can occur, which later in the course of operation of the equipment can lead to considerable material wear.

Former research into the tribocorrosion resistance of stainless steels has been carried out in several directions. Most of the research was carried out on tribometers (BALL-ON-DISC, PIN-ON-DISC, BLOCK-ON-DISC…) to determine the amount of wear and the friction factor at lower loads (5 to 50 N) and rotation speeds in the presence of acidic electrolytes 6 pH [3,4]. Industrial equipment must be operational in many chemical environments consisting of, or containing mixtures of, organic chemicals and acids, mineral acids, alkalis, salts, etc. Some of the most common acids used in the process industry are hydrochloric acid, phosphoric acid, nitric acid, sulfuric acid, and acetic acid. Hydrochloric acid is used to produce chloroacetic acid and many other organic compounds, while phosphoric acid is used in the production of artificial fertilizers. In the food industry, nitric acid is used to sterilize canned food, because it destroys bacteria that would otherwise spoil the food, and it is also widely used in chemical production. Sulfuric acid is often used as a wastewater treatment agent and is also used in chemical processes to produce carbon monoxide. The mentioned acids are widely used in many industries, from food to petrochemical to pharmaceutical [5].

The mechanical response of the tested stainless steels under tribocorrosion conditions is influenced by electrochemical conditions: both mechanical wear and wear-accelerated corrosion increase with the applied potential. In addition, delamination is the main wear mechanism that takes place under tribocorrosion conditions in the tested stainless steels. Accumulated deformation caused by sliding during tribocorrosive tests leads to low-cycle fatigue. Friction coefficients below 0.6 promote nanowear and higher wear rates are achieved when the friction coefficient increases [6]. In research conducted on special devices, the conclusions are approximately similar. The average passivation current density during tribocorrosion tests was significantly higher than the average current density where only corrosion resistance was tested. The average coefficient of friction in the tribocorrosion test was lower than the average coefficient of friction in the sliding wear tests. Apparently, the corrosion products formed during the tribocorrosion test acted as solid lubricants between the test specimen and the counter body. The synergistic effects of tribocorrosion are moderate. This result is attributed to the similarities between the wear mechanisms operating during tribocorrosion and sliding wear tests [1].

The real possibility of protecting machine parts in the process industry is certainly the appropriate choice of materials for tribosystem elements, as well as the choice of procedures for protecting surfaces from wear. In particular, stainless steels are chosen, and their mechanical properties and tribocorrosion resistance depend on the chemical composition and applied heat treatment. In the case of martensitic stainless steels, a good combination of mechanical properties was achieved by varying the temperature of austenitization and tempering and the duration of tempering. Corrosion current density and passivation current density vary with the temperature of austenitization. This variation can be attributed to the influence of some dissolved elements such as chromium, molybdenum, nickel, and carbon [7,8,9]. On the other hand, austenitic stainless steels are widely used due to their excellent corrosion resistance. The basic austenitic stainless steel is (AISI 304). It is an iron-based alloy containing nominally 18% chromium and 8.5% nickel, including smaller amounts of carbon, nitrogen, manganese, and silicon. A dozen new alloys have been developed from the basic (AISI 304) austenitic steel, based on the addition of, for example, molybdenum and nitrogen for better corrosion resistance. Regardless of the above, there are limitations in industrial application for this type of steel when exposed to different wear mechanisms. Most often, the aim of research is to reduce the wear intensity of austenitic stainless steels, using different thermochemical procedures [10,11,12].

Most authors analyze resistance to corrosion wear in a certain aggressive medium [13,14,15,16], and rarely with the presence of abrasive particles. Degradation of austenitic stainless steels in erosion-corrosion conditions is mainly determined by the mechanical action of impact particles, and in the case of martensitic stainless steels, it is accompanied by chemical action. In cases of simultaneous action of erosion and corrosion, the wear mechanisms are complex, and the measured mass loss is greater than the sum of the mass losses of the individual action of corrosion and erosion [17,18].

This paper presents an experimental investigation of the influence of load and rotation speed, as well as the aggressive effect of media on the resistance of modified surfaces of stainless steels in combined wear conditions.

## 2. Experimental Procedure

### 2.1. Materials and Surface Modification

For the purpose of conducting experimental tests, martensitic stainless steels X20Cr13 and X17CrNi16-2 were selected in a primary state. From round steel bars, samples of the dimensions and shapes required for the planned tests were machine-made. The spectrometric method was used to determine the chemical composition, and the tests were performed with the BELEC device. The chemical composition of the selected steels is shown in Table 1.

The heat treatment of martensitic steel samples was carried out by induction hardening as follows: frequency 19 kHz, energy 45%, austenitization temperature 1050 °C, emulsion quenching.

**Figure 1:**
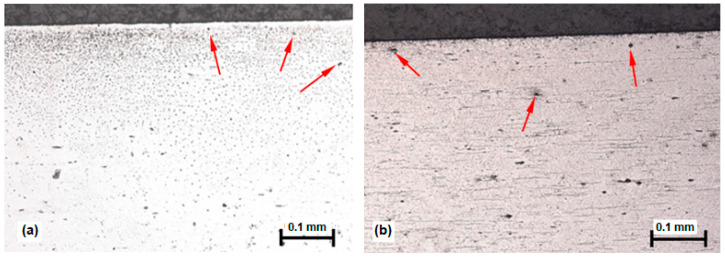
The microstructures of induction-hardened steels; (**a**) X20Cr13; (**b**) X17CrNi16-2.

Imaging of the microstructure was carried out on a Leica DM 2500 M light microscope (Leica, Wetzlar, Germany). The samples of martensitic stainless steels were etched with a solution of 10 mL HNO_3_ and 30 mL HCl in water. The Vickers Hv1 method was used to measure the microhardness of induction-hardened samples in a cross-section from the edge to the core. The mentioned tests were carried out on the DURIMET Leitz device (Leitz, Stuttgart, Germany). Analysis of the microstructures of the experimental materials (X20Cr13 and X17CrNi16-2) after modification of the surfaces by the induction hardening process shows a high concentration of secreted precipitates that decreases with distance from the surface (red arrows in Figure 1a,b), and the cores of both samples have a martensitic structure with dispersed carbides within the martensitic matrix.

The measured microhardness values of the induction-hardened X20Cr13 and X17CrNi16-2 steel samples, as well as the determined effective hardening depth (EHD), are shown in Table 2.

### 2.2. Testing Samples in Combined Wear Conditions

**Figure 2:**
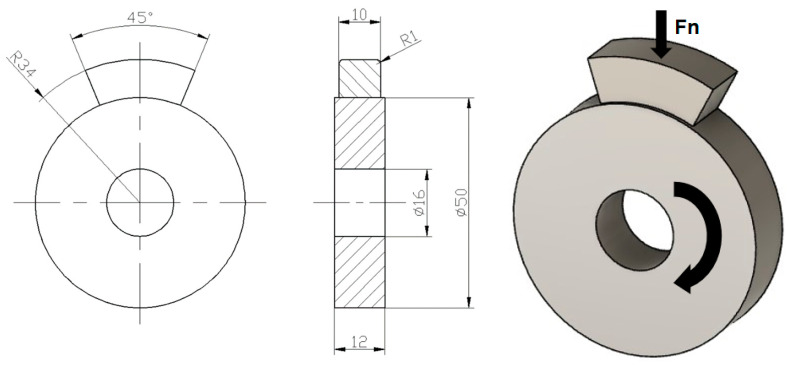
Test sample of resistance to wear in disc/counter body (slipper) contact.

Tests of resistance to combined wear are divided into two parts. The first part of the test of resistance to combined wear of the samples consisted of tests on the SMT-1 device for friction and wear control, in disc/counter body (slipper) contact (Figure 2), with the application of an aggressive medium in the chamber.

The contact between the surface of the disc sample and the counter body (slipper) is realized under conditions of sliding friction with the presence of microabrasive particles.

The test on the tribometer SMT-1 was carried out through two cycles of 11,460 revolutions per cycle. The austenitic stainless steel X2CrNiMo17-12-2, which has a basic chemical composition (wt%) (Table 1) was used as a counter body (slipper) for all test parameters. This steel was chosen because the use of similar materials is avoided when constructing parts exposed to adhesive wear [19,20,21] and because of its resistance to acids and intercrystalline corrosion [22].

As an aggressive test medium on the SMT-1 tribometer in the chamber, a heterogeneous mixture of standard quartz sand OTTAVA AFS 50/70 and a solution of acetic acid in distilled water with a volume concentration of 558 ppb was used, which corresponds to an acidity of pH 5. The amount of quartz sand was 10% by volume relative to the solution. The specified sand has a hardness of 1150 Hv, a density of 2150 kg/m^3^, and a fracture toughness of 1.6 MPa.

In order to create conditions for managing friction and wear processes in basic tribosystems, it is necessary to know the working parameters. The basic quantities used to identify the conditions for making contact are the speed of relative movement and the load at the point of contact [23].

Therefore, the goal of conducting the experiment is to determine the influence of operating parameters on the resistance to combined wear of modified sample surfaces. Since the test sample on which the output quantity (change in mass) is measured is a disk that rotates with the possibility of choosing the number of revolutions of the shaft, the first influencing factor will be the speed of rotation. Another influencing factor is the load on the upper sample that is at rest.

The test was carried out according to the factorial design of the experiment (3^2^) with two factors (load and rotation speed), each at three levels for each of the two materials. In order to make it clearer what the test conditions are, and for a clearer presentation of the wear resistance results, Table 3 shows the levels of the test parameters.

**Figure 3:**
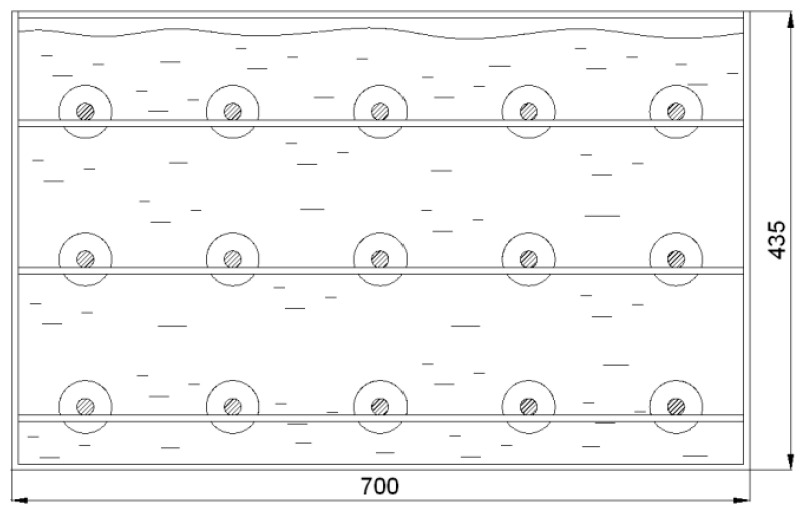
Arrangement of samples in a corrosion test bath.

In the second part of the test, in order to simulate the combined wear conditions in a time sequence, after each wear cycle of the samples on the tribometer, the exposure of these samples to the continuous action of the acetic acid solution in distilled water of pH 5 in a corrosion test bath was carried out (Figure 3).

The samples were placed 50 mm below the level of the solution, and they did not touch the wall and bottom of the container with the solution. The test was carried out at room temperature.

As an indicator of corrosion action and one of the basic parameters that shows the corrosion stability of metals is the corrosion rate, which is calculated according to Equation (1), where *m*_0_ is the mass before exposure to the corrosive environment, *m*_t_ is the mass after exposure to the corrosive environment, *ρ* is the material density, A is the area of the disc sample, and Δ*_t_* is the time interval:

(1) $$v_{\mathrm{corr}}=\frac{m_{0}-m_{t}}{A\cdot \;\Delta t\cdot \;\rho },\;[\mathrm{mm}/\mathrm{year}]$$

## 3. Results and Discussion

After testing the resistance to combined wear (on the SMT-1 tribometer and in a corrosion test bath), samples of martensitic stainless steels (X20Cr13 and X17CrNi16-2) were assessed and the weight of the discs was measured on an analytical balance (Kern ALS 220-4N, sensitivity 1 × 10^−4^ g).

The results of the mean mass loss for all three repetitions per test involving the induction-hardened steel X20Cr13 are presented in Table 4.

Based on the presented results of measuring the change in mass depending on the parameters of the tested samples, a certain dissipation of the measured values was observed. In order to determine the significance of the differences between the obtained results, statistical analysis was conducted using the licensed software Design Expert 9 [24,25].

The results of the mean mass loss for all three repetitions per test involving the induction-hardened steel X17CrNi16-2 are presented in Table 5.

### 3.1. Analysis of Variance for Steel X20Cr13

The analysis of the influence of load and rotation speed on the change in mass for hardened steel X20Cr13, and the verification of the significance of the model and its members, was made by analysis of variance (ANOVA), which is shown in Table 6. This analysis is also very common in experimental analyses, due to its effectiveness in interpreting the observed parameters [26]. The analysis proposed a rooting transformation with the addition of a constant term of 0.18062 in order to obtain positive values for mass change.

The *F*-value of the model is 27.13, which favors the selection of the model because the probability that such a large value appears due to noise is only 0.01%. Members with *p*-values less than 0.05 are significant for the variance of the output size. From Table 6, it can be seen that for the model the significant terms are rotation speed, B (*p* < 0.0001), the product of load and rotation speed, AB (*p* < 0.0001), the square of rotation speed, B^2^ (*p* = 0.0003), and the product of load and square of rotation speed AB^2^ (*p* = 0.0155). The obtained *p* value of 0.0603 for the impact of load is greater than the significance level of 0.05, but this member cannot be excluded from the model considering that it appears in member AB^2^ whose influence is significant in the model.

Moreover, in Table 6 it can be seen that the specified model is significant for the variance of mass change (*p* < 0.0001), which supports the selection of this model for the estimation of mass change. For the proposed response surface model, a mathematical model with coded factor values was developed, which is translated into a model with real factor values. Equation (1) shows the mathematical model of the dependence of the sample mass change on the load and rotation speed with the actual values of the factors for the X20Cr13 steel in the induction-hardened state.

(2) $$\Delta m=(-3.74502\;+\;0.017584\;\cdot \;F\;+\;0.015879\;\cdot \;n\;-\;5.50825\;{\cdot \;10}^{-5}\cdot F\cdot n\;-1.32649\;\cdot \;10^{-5}{\;\cdot \;n}^{2}+3.98479{\;\cdot \;10}^{-8}\;\cdot \;F{\;\cdot \;n}^{2})\;-\;0.18$$

**Figure 4:**
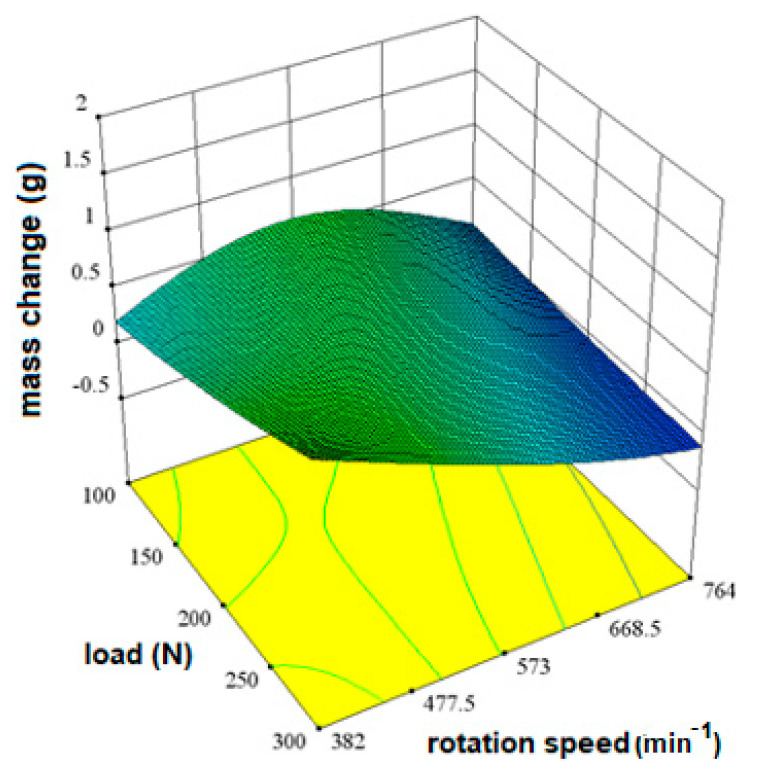
The influence of load and rotation speed on the change in disc mass for X20Cr13 steel as shown by a 3D response surface.

The influence of the load factor (A) and the rotation speed (B) can be shown graphically by observing the minimum and maximum value of each factor, as well as the amount of the output quantity. In this way, the tendency of the behavior of the output quantity with regard to the change of the input quantities is visible. Figure 4 shows the influence of factors on mass change.

Areas of greater mass change are shown in green and areas of lesser mass change are shown in blue. From the diagram in Figure 4, it can be seen that there are no high values shown in red. The shape of the response surface shows that the differences in the change are small and difficult to see, but as extreme values of the mass change, a load of 100 N at a rotation speed of 573 min^−1^ and values at a load of 300 N and a speed of 382 min^−1^ can be singled out.

### 3.2. Analysis of Variance for Steel X17CrNi16-2

The analysis of the influence of load and rotation speed on the mass change for X17CrNi16-2 steel in the induction-hardened state is presented in Table 7.

The variance analysis suggested a transformation of the type (y + k)^0.2^ with the addition of a constant term of 0.06 in order to obtain positive values for mass change.

The F-value of the model is 226.86, which favors the selection of the model because the probability that such a large value appears due to noise is only 0.01%. Members with *p*-values less than 0.05 are significant for the variance of the output size.

From Table 7, it can be seen that the significant terms for the model are the load, A (*p* < 0.0001), the speed of rotation, B (*p* < 0.0001), the product of the load and the speed of rotation, AB (*p* < 0.0001), the square of the load A^2^ (*p* = 0.0010), the square of the rotation speed, B^2^ (*p* < 0.0001), and the product of the square of the load and the rotation speed A^2^B (*p* = 0.0117). Moreover, in Table 7, it can be seen that the specified model is significant for the variance of mass change (*p* < 0.0001), which supports the selection of this model for the estimation of mass change.

For the proposed response surface model, a mathematical model with coded factor values was developed, which is translated into a model with real factor values. Equation (2) shows the mathematical model of the dependence of the sample mass change on the load and rotation speed with the actual values of the factors for the X17CrNi16-2 steel in the induction-hardened state.

(3) $$\Delta m=(-0.34008+8.79184\cdot 10^{-3}\cdot F+3.30292\cdot 10^{-3}\cdot n\;-\;1.31779\cdot 10^{-5}\cdot F\cdot n\;-1.56537\cdot 10^{-5}\cdot F^{2}\;-\;2.33581\cdot 10^{-6}\cdot n^{2}+1.97405\cdot 10^{-8}\cdot F^{2}\cdot n)\;-\;0.06$$

**Figure 5:**
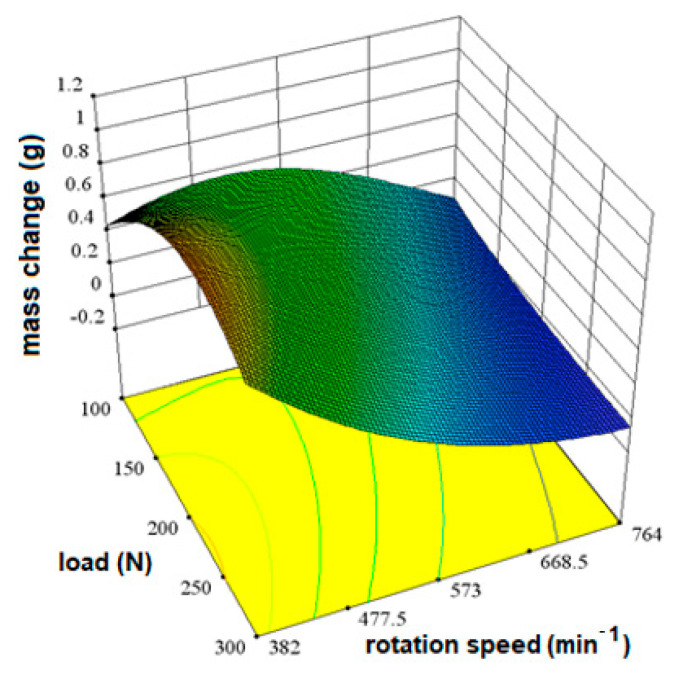
The influence of load and rotation speed on the change in disc mass for X17CrNi16-2 steel as shown by a 3D response surface.

Figure 5 shows the influence of factors on mass change. Areas of greatest mass change are shown in red, and areas of least change in blue.

From the graphic representation of the tendency of the behavior of the output size with respect to the change of the input sizes (Figure 5), it can be seen that the mass loss is minimal at the highest speed.

The region of the largest mass change is at a load of 200 N and a rotation speed of 382 min^−1^, while at the highest rotation speed a negligible loss of mass is noticeable regardless of the load.

### 3.3. Corrosion Test Analysis

Based on the presented results of mass loss measurements due to corrosion (Table 4 and Table 5), depending on the type of steel, a certain scattering of the measured values is also observed. In order to determine the significance of the differences between the obtained results, a statistical analysis of the data was performed using the licensed software Statistica 13.5.0.

Differences in mass loss values due to corrosion were tested for each individual condition using the Mann–Whitney U test. The Mann-Whitney U test is used to compare two independent groups of data when one dependent variable is ordinal or continuous and does not have a normal distribution. Table 8 shows the mean value, the standard deviation, and the median and interquartile range, as well as the *p* values of the Mann-Whitney U test.

From Table 8, it can be seen that there is no statistically significant difference by type of steel in the disc mass loss due to corrosion for the induction-hardened condition (Mann–Whitney U test, *p* = 0.709841).

### 3.4. Interaction Effect of Combined Wear

**Figure 6:**
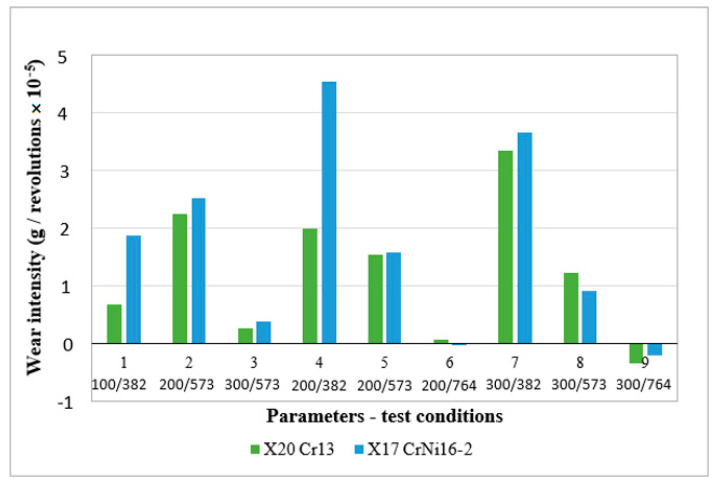
Dependence of wear intensity of tested steel samples.

In order to see the interaction effect due to the wear on the tribometer and the corrosion effect in the bath for corrosion testing, a quantitative presentation of the average wear intensity values of the tested samples was made. These results were calculated on the basis of the mean wear intensity values from Table 4 and Table 5 and are shown in a histogram in Figure 6.

Observing on the *x*-axis of the histogram all test conditions (parameter combinations) for both induction-hardened steels, higher wear resistance was achieved by steel X20Cr13 in conditions one, two, three, four, and seven with an average value of wear intensity I_wX20Cr13_ = 1.22 × 10^−5^ in all conditions.

Steel X17CrNi16-2 had higher resistance to wear only in conditions six, eight, and nine. In other conditions, it wore more significantly with an average intensity of wear I_wX17CrNi16-2_ = 1.68 × 10^−5^, compared to steel X20Cr13, which proved to be more resistant.

### 3.5. Wear Traces Analysis

After the combined wear test (on tribometer and in a corrosion test bath), wear marks were recorded on working surfaces of the discs.

**Figure 7:**
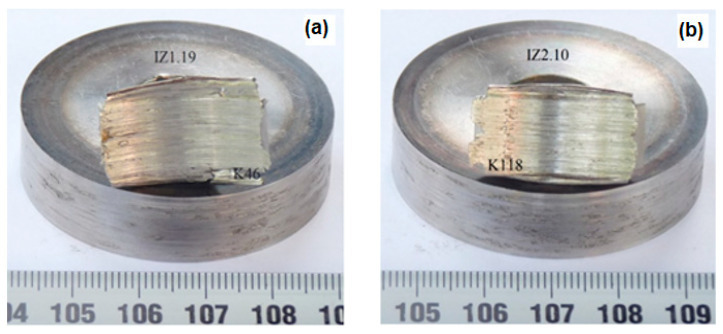
Macro view of the wear surfaces of tribopair of hardened steel samples; (**a**) X20Cr13; (**b**) X17CrNi16-2.

Figure 7 shows the macro view of the worn surfaces of the disc/slipper tribopair test samples for X20Cr13 and X17CrNi16-2 steel in the induction-hardened state, which had the highest mass loss after wear on the SMT-1 tribometer.

**Figure 8:**
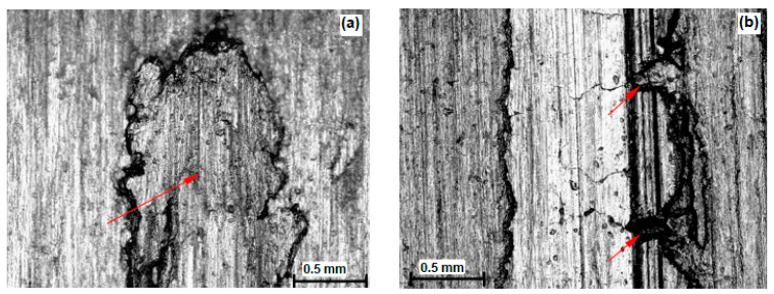
Traces of abrasive and adhesive wear of hardened steel X20Cr13; (**a**) sample IZ 1.16; (**b**) sample IZ1.19.

In Figure 8a, for disc sample IZ 1.19 with steel X20Cr13, a particle can just before be seen falling out of the base material (red arrow in the picture) as a result of adhesive wear.

In Figure 8b, an area of wear marks characteristic of selective abrasion can be observed, where the discontinuity of the plough can be observed in the places of the remaining harder structural constituents (red arrows in the picture).

**Figure 9:**
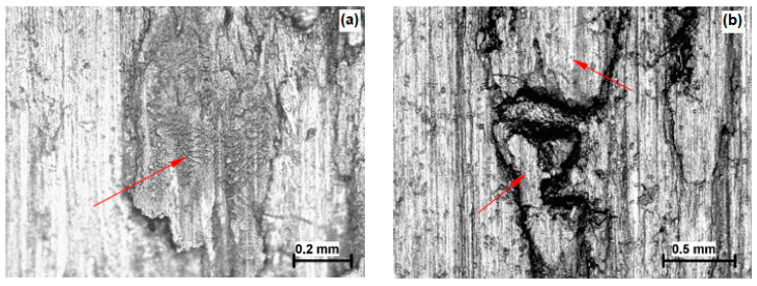
Detail of the worn surface of the disc sample IZ 2.10 by the adhesive wear mechanism for steel X17CrNi16-2; (**a**) sticking of the material; (**b**) particle directly before falling out.

Figure 9a,b shows a detail of the worn surface caused by adhesion on the disc sample IZ 2.10 with steel X17CrNi16-2 in the induction-hardened state.

In Figure 9a, the transition of material from one friction surface to another in direct contact conditions is observed, in this case the transition from a softer counter body to a harder disc.

In Figure 9b, a particle can be seen just before falling out followed by a crater in the place where carbide loss occurred (red arrows in the picture).

**Figure 10:**
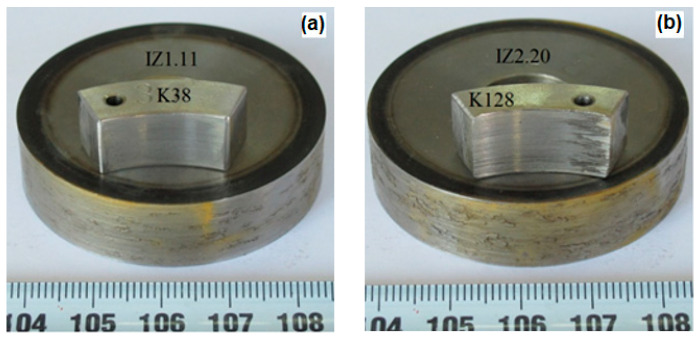
Macro view of the corroded surfaces of tribopair of hardened steel samples; (**a**) X20Cr13; (**b**) X17CrNi16-2.

Figure 10 shows the macro view of the corroded surfaces of the disc/counter body tribopair test samples after they were exposed to the continuous seven-day action of the aggressive medium in the bath. These are induction-hardened disc marked IZ 1.11, steel X20Cr13 and induction-hardened disc marked IZ 2.20, steel X17CrNi16-2.

**Figure 11:**
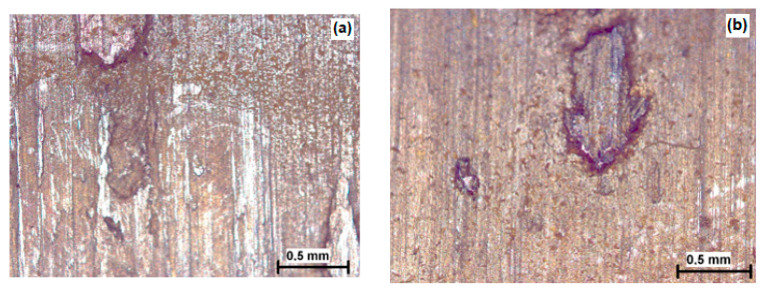
Corrosion products on the surfaces of disc samples; (**a**) X20Cr13; (**b**) X17CrNi16-2.

Figure 11a,b shows the appearance of the corroded surfaces of test samples of discs in the induction-hardened state of IZ 1.11 steel X20Cr13 and IZ 2.20 steel X17CrNi16-2.

Corrosion products on the surfaces of the discs for both steels after the action of the aggressive medium in the bath are not very pronounced. Underneath the corrosion marks, the shine of the base material can be seen.

## 4. Conclusions

Based on this research concerning the influence of rotation speed and load on the resistance of the hardened surfaces of martensitic stainless steels in tribocorrosive wear conditions, several conclusions were reached.

Statistical processing of the obtained data showed a significant influence of rotation speed and load. Further processing of the data by regression analysis resulted in mathematical models that exactly define the interrelations of the influencing parameters and correspond very well to the experimental data. Furthermore, the results of this research showed several significant combinations of load and rotation speed where the least wear was achieved. The most favorable combination of parameters for steel in the hardened state is the combination of 200 N/764 min^−1^ for X20Cr13 steel, and 100 N/764 min^−1^ for X17CrNi16-2 steel.

After analyzing the results of the corrosion resistance test using the gravimetric method, no statistically significant influence of corrosion on the mass loss of the test samples was determined. If the average values of corrosion rates are compared, the induction-hardened steel X17CrNi16-2 compared to X20Cr13 has a 9% lower corrosion rate. Better anti-corrosion properties of steel X17CrNi16-2 are found in the higher content of chromium and nickel, which additionally increase the corrosion resistance.

The comparison of the intensity of combined wear showed greater resistance to combined wear in X20Cr13 steel, which had a 27% lower wear intensity compared to X17CrNi16-2 steel. The higher wear resistance achieved can be attributed to the higher surface hardness and effective hardening depth of 2.25 mm in X20Cr13 steel compared to 1.25 mm in X17CrNi16-2 steel.

The mentioned resistance is the result of the creation of a martensitic surface layer with dispersed carbides, which increases the resistance to abrasion, dynamic durability, and fatigue of the surface of the protective layer. The applied procedure for surfaces modification of martensitic stainless steels provides resistance in combined wear conditions, where there is a synergistic effect of mechanical wear and the aggressive action of an acidic medium.

## Figures and Tables

**Table 1:** The chemical composition of steel test samples (wt%).

Steel	% C	% Mn	% Si	% Cr	% Ni	% Mo
X20Cr13	0.20	0.46	0.32	12.98	0.20	0.06
X17CrNi16-2	0.22	0.42	0.43	15.93	2.03	0.03
X2CrNiMo17-12-2	0.02	1.49	0.53	17.06	10.22	2.44

**Table 2:** The results of microhardness measurements of hardened samples.

Steel	Hv1	EHD (mm)
X20Cr13	610	2.25
X17CrNi16-2	533	1.25

**Table 3:** Parameters of the wear resistance test on the SMT-1 tribometer.

Parameter Description	Unit	Parameter Levels		
Normal sample load, *Fn*	(N)	100	200	300
Rotation speed of the disc pattern, *v*	(min^−1^)	382	573	764

**Table 4:** Test results of resistance to combined wear, hardened steel X20Cr13.

Steel	Test Parameters		Mean Mass Loss, g		Wear Intensity, *I*_w_ (g/rev.)	Corrosion Rate, *v*_corr_ (mm/year)
			On SMT-1 (Δ*m*_device_)	In the Bath (Δ*m*_corrosion_)		
	*F_n_*, (N)	(*v*, min^−1^)				
X20Cr13	100	382	0.145	0.0088	6.71 × 10^−6^	4.99 × 10^−3^
	100	573	0.5035	0.0110	2.24 × 10^−5^	6.19 × 10^−3^
	100	764	0.0427	0.0179	2.65 × 10^−6^	1.01 × 10^−2^
	200	382	0.4391	0.0153	1.98 × 10^−5^	8.63 × 10^−3^
	200	573	0.3431	0.0104	1.54 × 10^−5^	5.86 × 10^−3^
	200	764	0.0062	0.0085	6.43 × 10^−7^	4.81 × 10^−3^
	300	382	0.7600	0.0063	3.34 × 10^−5^	3.57 × 10^−3^
	300	573	0.2783	0.0012	1.22 × 10^−5^	7.13 × 10^−4^
	300	764	−0.0901	0.0085	−3.56 × 10^−6^	4.81 × 10^−3^

**Table 5:** Test results of resistance to combined wear, hardened steel X17CrNi16-2.

Steel	Test Parameters		Mean Mass Loss, g		Wear Intensity, *I*_w_ (g/rev.)	Corrosion Rate, *v*_corr_ (mm/year)
			On SMT-1 (Δ*m*_device_)	In the Bath (Δ*m*_corrosion_)		
	*F_n_*, (N)	(*v*, min^−1^)				
X17CrNi16-2	100	382	0.4267	0.0014	1.86 × 10^−5^	7.88 × 10^−4^
	100	573	0.5486	0.0282	2.51 × 10^−5^	1.59 × 10^−2^
	100	764	0.0761	0.0084	3.68 × 10^−6^	4.77 × 10^−3^
	200	382	1.029	0.0107	4.53 × 10^−5^	6.04 × 10^−3^
	200	573	0.3445	0.0142	1.56 × 10^−5^	8.03 × 10^−3^
	200	764	−0.0166	0.0082	−3.66 × 10^−7^	4.62 × 10^−3^
	300	382	0.8223	0.0108	3.64 × 10^−5^	6.12 × 10^−3^
	300	573	0.2038	0.0052	9.12 × 10^−6^	2.97 × 10^−3^
	300	764	−0.063	0.0120	−2.22 × 10^−6^	6.80 × 10^−3^

**Table 6:** ANOVA of the selected model for induction-hardened steel X20Cr13.

ANOVA for Response Surface Reduced Cubic Model Transformation: Type Square Root, Constant 0.18062						
Source	Sum of Squares	DF	Mean Square	F Value	*p*-Value Prob ˃ F	Significance
Model	0.048	5	0.33	27.13	˂0.0001	significant
A-load	0.048	1	0.048	3.94	0.0603	not significant
B-rotation speed	0.95	1	0.95	78.39	˂0.0001	significant
A·B	0.39	1	0.39	31.87	˂0.0001	significant
B^2^	0.22	1	0.22	18.38	0.0003	significant
A^2^·B	0.085	1	0.085	66.94	0.0155	significant
Residual	0.26	21	0.012			
Lack of fit	0.013	3	4.24 × 10^−3^	0.31	0.8147	not significant
Pure Error	0.24	18	0.014			
Cor Total	1.91	26				

**Table 7:** ANOVA of the selected model for induction-hardened steel X17CrNi16-2.

ANOVA for Response Surface Reduced Cubic Model Transformation: Type (y + k)^0.2^, Constant 0.06						
Source	Sum of Squares	DF	Mean Square	F Value	*p*-Value Prob ˃ F	Significance
Model	1.00	6	0.17	226.86	˂0.0001	significant
A-load	0.041	1	0.041	56.67	˂0.0001	significant
B-rotation speed	0.33	1	0.33	445.18	˂0.0001	significant
A·B	0.12	1	0.12	166.91	˂0.0001	significant
A^2^	0.011	1	0.011	15.12	˂0.0001	significant
B^2^	0.040	1	0.040	54.48	˂0.0001	significant
A^2^·B	5.68 × 10^−3^	1	5.68 × 10^−3^	7.77	0.0117	significant
Residual	0.014	19	7.31 × 10^−6^			
Lack of fit	4.12 × 10^−3^	2	2.06 × 10^−3^	3.58	0.0503	not significant
Pure Error	9.77 × 10^−3^	14	5.75 × 10^−4^			
Cor Total	1.01	25				

**Table 8:** Disc mass loss values due to corrosion depending on the steel.

State/Steel	Value of Disc Mass Loss in Grams		*p* ^§^
Induction-Hardened	SD ^†^	Median (25–75%)	
X20Cr13	0.0098 (±0.0047)	0.0960 (0.0074–0.0122)	0.709841
X17CrNi16-2	0.0110 (±0.0077)	0.0098 (0.0072–0.0126)	

^†^ standard deviation, ^§^ Mann-Whitney U test.

## Data Availability

Not applicable.
